# Development and evaluation of a website with patients experiences of multiple sclerosis: a mixed methods study

**DOI:** 10.1186/s12883-022-02663-9

**Published:** 2022-04-20

**Authors:** Anna Sippel, Jutta Scheiderbauer, Désirée Eklund, Sigrid Arnade, Stephan Schmidt, Ingo Kleiter, Rebecca Morrison, Christopher Kofahl, Christoph Heesen

**Affiliations:** 1 Institute of Neuroimmunology and Multiple Sclerosis (INIMS), University Medical Center Hamburg-Eppendorf (UKE), Martinistrasse 52, 20246 Hamburg, Germany; 2 Trier, Germany; 3 LEBENSNERV – Stiftung zur Förderung der psychosomatischen MS-Forschung, Berlin, Germany; 4 Neurologische Gemeinschaftspraxis, Gesundheitszentrum St. Johannes, Bonn, Germany; 5 Marianne-Strauß-Klinik, Behandlungszentrum Kempfenhausen für Multiple Sklerose Kranke gGmbH, Berg, Germany; 6 St. Josef-Hospital, Department of Neurology, Ruhr-University Bochum, Bochum, Germany; 7 Berlin, Germany; 8 Institute of Medical Sociology, University Medical Center Hamburg-Eppendorf (UKE), Hamburg, Germany; 9 Department of Neurology, University Medical Center Hamburg-Eppendorf (UKE), Hamburg, Germany

**Keywords:** Multiple sclerosis, Decision making, Web-based experiential information, Patient experiences

## Abstract

**Background:**

A variety of management options (e.g., disease-modifying therapy, lifestyle interventions, rehabilitation) are available for persons with relapsing-remitting multiple sclerosis (MS). Besides coping with the diagnosis, persons with MS have to make complex decisions, e.g., regarding disease-modifying therapies. In addition to factual information, reports of patient experiences may support other patients in their decision-making. Therefore, we developed a website presenting patient experiences illustrated by video, audio and text files. This study aimed to test the acceptability and usability of a website with patient experiences with MS.

**Methods:**

A mixed-methods approach was applied. A total of 69 participants visited the German “Patient Experiences with MS (PExMS)” website and among them, 50 persons with MS and 6 experts completed an online survey. In total, 18 participants took part in telephone interviews or focus groups. Data from the survey were analysed using descriptive statistics. Qualitative data were analysed using thematic analysis.

**Results:**

Both quantitative and qualitative responses suggest that the PExMS website was viewed positively by patients and experts. 94% of persons with MS agreed that the information was comprehensible and reliable. 54% felt encouraged to share their health problems with others after having studied the website. 74% claimed to use the website if they had to make a decision regarding their health. Qualitative responses deduced from the website fell into 5 key themes: (1) web design, appearance, and functionality, (2) content, (3) usability, (4) satisfaction, and (5) loyalty. The search for persons of similar age and with comparable experiences was a major driving force to navigate the website. The material on the website was perceived as diverse, covering both positive and negative experiences in daily living with MS. All participants greatly appreciated having access to other people’s experiences online and judged the material on the website as particularly helpful in decision-making for disease-modifying therapies.

**Conclusions:**

The findings suggest that the PExMS website might have the potential to be a useful source of audio-visual information for persons with MS. Given the lack of websites available to patients with experiential information, health care professionals may be encouraged to routinely inform patients about this website at regular appointments.

**Supplementary Information:**

The online version contains supplementary material available at 10.1186/s12883-022-02663-9.

## Background

Multiple sclerosis (MS) is a chronic, inflammatory, and degenerative disease of the central nervous system. In Germany, there are about 250.000 persons with MS, mostly diagnosed between age 20 and 40. Females are affected by MS three times more often than males [1]. Three disease courses are differentiated: (1) Relapsing-remitting MS (RRMS) is most common, characterized by unpredictable relapses followed by transient periods with no new signs of disease activity. (2) Approximately half of the patients with RRMS have transitioned to secondary progressive MS (SPMS) 15 years after the diagnosis, and (3) about 10–15% are diagnosed with primary progressive MS (PPMS) [2].

In the last two decades, a wide range of disease-modifying therapies (DMTs) for RRMS have become available and novel treatments continue to emerge. DMTs aim to reduce the number and severity of inflammatory attacks and to delay transition to the progressive phase of MS. All of them can have mild to severe adverse effects. Although data on the long-term effectiveness of DMTs are scarce [3], data from registries indicate that early treatment with DMTs reduces the risk for progression [4, 5]. Besides, patients use other approaches such as lifestyle interventions, rehabilitation, and complementary alternative therapies [6]. The choice of the most appropriate therapy for the individual person requires the consideration and involvement of multiple factors, which is a constant challenge to both patients and physicians [7–10].

Confronted with new health problems or treatment decisions, persons with MS most frequently seek information on the internet prior to and after doctor’s visits [11]. Patients do not only search for evidence-based information but also for other patients´ experiences, online and in-person [10, 11]. There is a limited but increasing body of research addressing the value of websites featuring patients’ experiences [10, 12–22], especially with regard to their effect on decision-making [15, 21]. Therefore, there is a need for care-oriented and high-quality research to make patients´ experiences accessible and to study their effect [23]. We addressed this issue by developing a multi-media website of patient experiences with MS.

Evaluation of this web-based resource with patients´ experiences is important to ensure providing support for persons with MS in decision-making on any DMT. Therefore, the aim of this study was to examine the web design, appearance and functionality, content, usability, and the satisfaction as well as the impact on patients after having visited the website. The latter contains the extent to which patients gain confidence in discussing their health; the extent to which patients feel they better understand their health condition, how much they feel reassured, and motivated to manage their health and if their self-confidence in decision-making is enhanced.

## Methods

### Website development

This study is part of the project “Patient Experiences of Multiple Sclerosis (PExMS)” [24], aiming to evaluate whether patients’ experiences may help other persons with MS in their decision-making process regarding a possible treatment with a DMT, supplementary to evidence-based information. The project is monitored by a six-member advisory panel (representatives of persons with MS, researchers and neurologists) and follows the guidance of the Medical Research Council [25] for the development and evaluation of complex interventions. The process has a development phase as well as a feasibility and a pilot phase, with the latter still pending. The development phase is reported according to the ‘Consolidated Standards of Reporting Trials of Electronic and Mobile HEalth Applications and onLine TeleHealth’ (CONSORT-EHEALTH) [26].

**Fig. 1:**
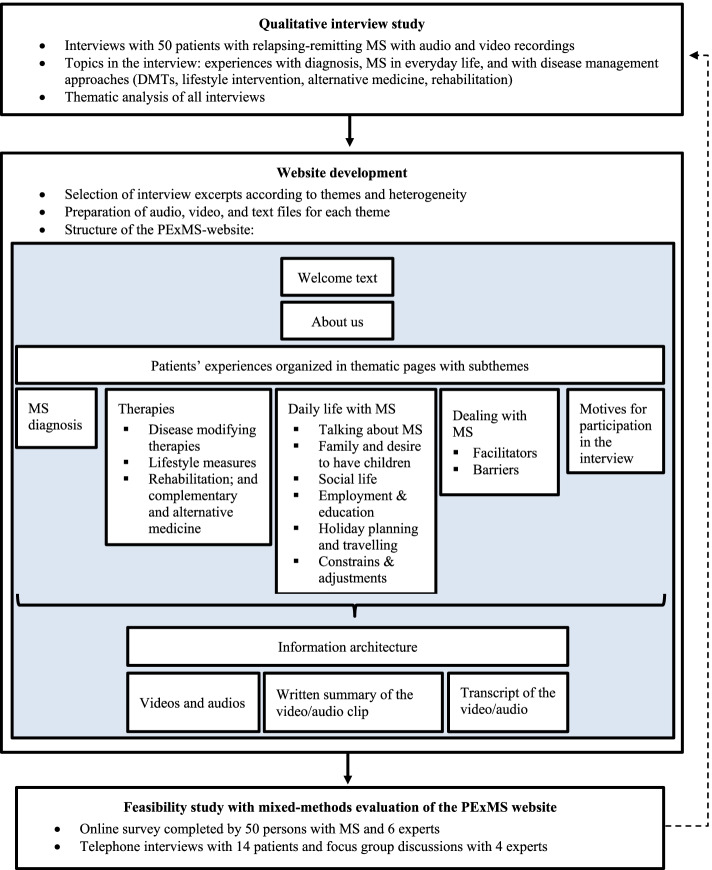
Development process and structure of the PExMS website

In the development phase (Fig. 1), experiences with MS were systematically collected from 50 patients with RRMS via interviews using the maximum variation sampling method [27]. The concept followed the approach of sharing patients’ experiences of the ‘Database of Individual Patients’ Experiences’ (DIPEx) [28–30], though it was not a formal DIPEx project. The interviews were performed in people’s homes, in clinics, in a rehabilitation centre or other places of their choice and were audio- and video-recorded. Afterwards, they were transcribed and thematically analysed according to Braun and Clarke [31]. Different themes related to experiences with MS in daily life and with therapies were identified in the dataset, analysed, organised, and described (for more information on the methods and results of the interview study see [24, 32]).

To generate content for the website, interview extracts were selected from the transcripts for each theme, in particular with regard to different sexes, ages and experiences from different point of views. The aim was to provide an appropriate balance of different patient experiences on the PExMS-website.

Then, audio and video clips as well as summaries written in lay language and transcripts of the audio and video clips were edited. The structure and the different themes of the PExMS-website called ‘ms-erfahrungen’ are shown in Fig. 1. See Additional file 1 as an example of website contents.

Moreover, the accuracy of all medical information provided in the narratives was examined by the principal investigators of this study (AS and CH) and the advisory panel. If necessary, obvious false factual information in the videos and audios has been omitted. Besides, all persons appearing in the videos and audios were given pseudonyms and all personal references to the narrators themselves or to other institutions, medical staff, etc. were deleted.

Before the launch of the website, participants of the qualitative study were given transcriptions of the selected media files and access to the preliminary version of the website and were asked again for permission for uploading. The website contained 37 subtopics and 336 media files organized into five main topics. However, no evidence-based information or links to other websites can be found on the PExMS-website so far.

### Study design

In this feasibility study, a mixed-methods approach was used to collect both qualitative and quantitative data using an online survey and telephone interviews or focus groups.

### Participants

Participants were German-speaking persons with possible or definite MS, at least 18 years of age, and with internet access. Recruitment took place between December 2020 and February 2021 via newsletters of the MS day hospital at the University Medical Center Hamburg-Eppendorf and during medical appointments. Moreover, experts from the advisory panel including representatives of persons with MS, researchers, and neurologists were involved.

### Data collection

#### Online survey

Participants were invited to perform an informal heuristic evaluation of the website, with particular focus on the design of the website, the suitability of the material, ease of navigation, perceived reliability, and participants’ general level of engagement (number of logins and time spend on the website). As the PExMS website was not publicly available, participants were granted access via email for a testing phase of 2–3 weeks (access to the website, see Additional file 2). Having tested the website, participants were invited to access an anonymous online survey by an electronic link. Having obtained informed consent, patients were asked to provide information on the following matters:

- socio-demographic and MS-related questionnaires (16 items), such as the 9-item-Patient Determined Disease Steps (PDDS), which asks for the patient-reported walking ability and disability [33]
- 15-item process evaluation questionnaire (Additional file 3)
- self-confidence in decision-making: 11-item Decision Self-Efficacy Scale (DSES) [34, 35]
- the usefulness of the PExMS website in preparing to communicate at a consultation visit and to decide on a therapy: 10-item Preparation for Decision Making (PrepDM) scale [25, 36]
- decisional conflict: 4-item SURE (Sure of myself; Understand information; Risk-benefit ratio; Encouragement) scale [37]
- confidence in active participation and management in medical care: 13-item Patient Activation Measure 13 (PAM13-D) [38]
- general attitudes towards using the internet to gain health information and attitudes towards the PExMS website: 11-item eHealth Impact Questionnaire (eHIQ) part 1 and 26-item eHIQ part 2 [39, 40]

By contrast, experts were only asked to fill out socio-demographic (4 items) and process evaluation questionnaires (14 items) (Additional file 3).

#### Telephone interviews and focus group discussions

At the end of the survey, participants were asked to take part in a short interview and to provide contact details on request. One-to-one telephone interviews with persons with MS and online focus group discussions with experts consisting of representatives of persons with MS, researchers and neurologists were conducted. The interviewer and moderator was AS, a health scientist experienced in qualitative research. Based on a website evaluation guide [41], participants were asked to provide their experiences with the website: 1) general impression, 2) web design and appearance, 3) content, and 4) suggestions for improvement (see Table 1). Interviews and focus groups were audio-recorded and transcribed verbatim for analysis. For ethical reasons, all participants were given a participant number, and all quotations used in the result section have been cleared of any information that could potentially reveal the identity of the participant.

**Table 1:** Interview guide for experts and patients with MS on their experiences with the PExMS website

Interview guide	
1. What is your overall impression of the website? - What did you particularly like about the website and why? - What did you particularly not like about the website and why? 2. What is your impression of the design and appearance of the website? - Layout - Colour scheme - Graphical elements - Image and sound quality of the videos and audios 3. What is your impression of the content of the website? - Are the topics in the videos and audios presented in a balanced way? - Is any important topic not represented on the website? - Were there any video or audio clips that irritated you and if so, why? - For what purposes would you use the website? 4. What would you like to see in the further development of the website? 5. Is there anything you would like to add that hasn’t been addressed yet?	

#### Data analysis

Survey data were analysed descriptively using SPSS (version 25.0; IBM Corp.). Continuous variables are described using mean and standard deviation (SD) and categorical items are presented as numbers and percentages.

All verbalized data from the focus groups and telephone interviews were audio-recorded for verbatim transcription. Afterwards, qualitative data were analysed thematically [31] by using the framework proposed by Allison et al. (2019), who suggest to evaluate specific website attributes, such as usability, content, web design, and functionality [41]. These data were processed using MAXQDA Analytics Pro 2018.

## Results

### Quantitative results

#### Sample description

Six experts from the advisory panel visited the website and completed the assigned questionnaire. 50% of them were females, with a mean age of 55 years (SD = 5). MS expertise consisted of being a researcher (*N* = 2), a neurologist (*N* = 1), and/or being a representative of patient groups (*N* = 4).

From the total number of participants with MS (*N* = 63) who got access to the website and the online survey, 50 patients (79%) completed the questionnaire (Table 2). Most of them were female (72%), with a mean age of 48 years. There was a high level of education with 70% having achieved a high school degree. Nearly all participants had a definite MS diagnosis, while 8% had a possible MS having a clinically isolated syndrome. 68% of those with definite MS had a relapsing-remitting MS course. The patients had a mean duration of MS of 12 years and a mean PDDS of 2 (moderate disability) [33]. Regarding the stage of the decision-making process for a DMT, 40% had already made a decision and were unlikely to change their mind, while 52% (*N* = 26) were still thinking about different DMT options. The mean score for patients’ general attitudes towards using the Internet to access health information and their attitude at using (experiential) online information for learning and gaining support (eHIQ part 1) was average. 28% reported that they had already taken part in the qualitative interview study in the development phase of the website and were thus personally displayed on the website.

**Table 2:** Demographics of persons with MS (*N* = 50)

Characteristics	Patients
Women, n (%)	36 (72)
Age in years, mean (SD)	48 (13)
Education, n (%)	
Primary degree (9 grades)	3 (6)
Secondary degree (10 grades)	12 (24)
High school degree (12/13 grades)	35 (70)
MS diagnosis, n (%)	
Suspicion of MS	4 (8)
MS diagnosis	46 (92)
Years with MS since diagnosis, mean (SD)	12 (10)
MS type, n (%)	
Clinically isolated syndrome	1 (2)
Relapsing-remitting MS	34 (68)
Secondary-progressive MS	9 (18)
Primary-progressive MS	4 (8)
Unclear	2 (4)
Patient determined disease steps (PDDS), mean (SD)	2 (2)
Stage of Decision Making in DMT, n (%)	
Persons haven’t begun to think about the choices	4 (8)
Persons haven’t begun to think about the choices, but are interested in doing so	1 (2)
Persons are considering the options now	4 (8)
Persons are close to selecting an option	4 (8)
Persons have already made a decision, but are still willing to reconsider	17 (34)
Persons have already made a decision and are unlikely to change their mind	20 (40)
eHIQ part 1 sum index score, mean (SD)	58.2 (18.2)
1.1 attitudes towards online health information	52.0 (20.4)
1.2 attitudes towards sharing health experiences online	64.3 (19.1)
Participants from qual. Interview study in development phase, n (%)	14 (28)

#### Navigation, usefulness and satisfaction

In the 3-week testing phase, patients spend on average 3.2 h and experts 2.5 h browsing the website. Patients most often viewed the main themes “MS diagnosis”, “DMT” and “Talking about MS with others”. Participants filtered for age and sex when choosing a video or audio clip, with age having descriptively a stronger influence (mean = 3.3) on the users than sex (mean = 4.4) on a six-point Likert scale (Table 3).

**Table 3:** User’s navigation and usefulness of the website (*N* = 50)

Characteristics	Patients
Number of logins, n (%)	
More than 1 time a week, n (%)	7 (14)
Once a week at most	26 (52)
Once a month at most	16 (32)
Spent time (in hours), mean (SD)	3.2 (4.3)
Influence of age on choosing a video/audio ^a^, mean (SD)	3.3 (1.6)
Influence of sex on choosing a video/audio ^a^, mean (SD)	4.4 (1.4)
Themes browsed, n (%)	
MS diagnosis	40 (80)
Disease-modifying therapies	40 (80)
Lifestyle measures	36 (72)
Rehabilitation	35 (70)
Talking about MS with others	40 (80)
Family and desire for children	19 (38)
Social life	32 (64)
Training and work	27 (54)
Holiday planning and travelling	17 (34)
Constrains and adaptions	32 (64)
Coping with MS	38 (76)
eHIQ part 2 sum index score, mean (SD)	70.7 (12.9)
2.1 confidence and identification	67.4 (15.1)
2.2 information and presentation	81.8 (12.9)
2.3 understanding and motivation	62.9 (16.7)
Grade for the website ^b^, mean (SD)	2.1 (0.8)
Helpful in DMT decision-making ^c^, mean (SD)	2.3 (0.8)
SURE ^d^, mean (SD)	4.4 (1.6)
DSES ^c^, mean (SD)	83.9 (16.0)
PrepDM ^f^, mean (SD)	53.3 (27.1)
PAM ^g^, mean (SD)	42.3 (4.5)

^a^ Scale range from 1 (very strong influence) – 6 (no influence at all). ^b^ Scale range from 1 (best possible grade) – 6 (lowest possible grade). ^c^ Scale range from 1 (very helpful) – 5 (not helpful at all). ^d^ A score of < 4 indicates a perception of a decisional conflict. ^e^ Higher scores indicate higher perceived level of self-efficacy. ^f^ Higher scores indicate higher perceived level of preparation for decision-making. ^g^ The sum score range from 13 to 52. Higher scores indicate higher perceived level of knowledge, skill, and confidence in managing chronic conditions

**Fig. 2:**
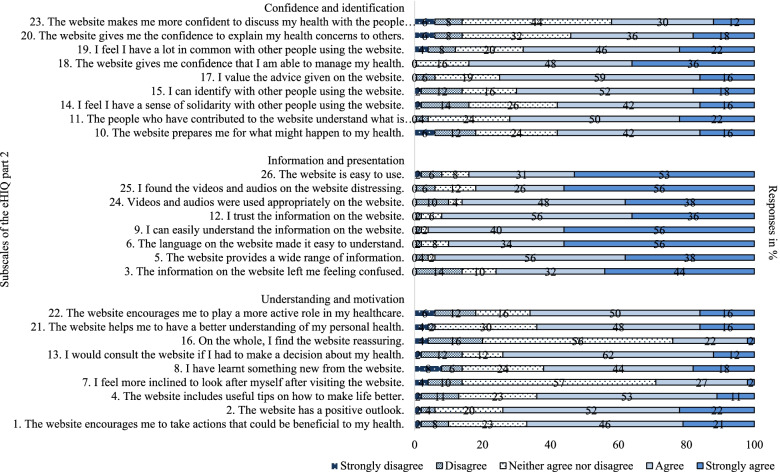
eHIQ part 2 - patients’ views about the PExMS website

The website reached a sum index score on the eHIQ part 2 of 70.7 on a scale ranging from 0 to 100. The highest score was achieved in the subscale ‘information and presentation’, which reflects users’ trust and suitability of the website content (Table 3). 84% agreed or strongly agreed that the website is easy to use, and 94% agreed or strongly agreed that the information was easy to understand and trustworthy. 86% perceived that the videos and audios were used appropriately on the website. However, 84% of the patients expressed feelings of distress and 76% felt confused (Fig. 2).

Participants’ confidence to discuss their health condition with others after browsing the website and their ability to identify themselves with the users of the website (eHIQ 2 ‘confidence and identification’) was moderate (Table 3). Approximately 70% were able to identify themselves and felt a lot in common with the experience of other patients using the website. Half of the patients felt encouraged to discuss their health issues with their personal surroundings and to share them with fellow patients (Fig. 2).

The score in the subscale ‘understanding and motivation’, which reflects the understanding and learning about relevant health information and motivation to act was moderate as well (Table 3). 74% agreed or strongly agreed that they would consult the website if they had to make a decision concerning their health. More than half of the participants stated that the website encouraged them to take actions potentially beneficial to their health and to play a more active role in their personal healthcare. 56% neither agreed nor disagreed that the website is reassuring (Fig. 2).

An exploratory analysis of the influence of MS and demographic variables (age, sex, the stage of decision-making for a DMT) on outcomes related to user’s navigation and the usefulness of the website showed no significant impact.

Participants rated the website as helpful when deciding for a DMT (Table 3). We examined the patients’ decisional conflict (SURE) and decision self-efficacy after viewing the website (DSES). Persons with MS experienced no decisional conflict and felt confident in making informed choices about their treatment. Patients’ perception of the website’s usefulness for preparing them for their next doctor’s visit and for making a health decision (PrepDM) was moderate. However, the perceived level of knowledge, skill, and confidence in managing MS was high (PAM). Taken together, the website was evaluated positively and therefore graded with 2.1 (SD = 0.8) by patients and 1.7 (SD = 0.5) by experts on a scale from 1 (excellent) to 6 (very poor).

### Qualitative results

Fourteen persons with MS and four experts, with three-quarters of females in each group, took part in the follow-up telephone interviews or in the two focus groups.

We analysed the interview and focus group data with the framework for website evaluation [41] using the following website attributes: web design, appearance, functionality, content, usability, satisfaction, and loyalty. These attributes are illustrated in Additional file 4 using quotes as examples of negative and positive feedback.

#### Web design, appearance and functionality

Web design criteria include for example the site map, use of media, and search engines [41]. Participants were very satisfied with the web design, especially appreciating the different forms of media including videos, audios and written material.

*“I perceived the way it was put together very modern, the pictures and the choice of images, all very appropriate.” (person with MS 41)*

At present, there is no possibility to leave feedback or comments on the website visible for all users. For a few participants, the existence of a web forum or some other means by which users could contact the protagonists of the website directly (i.e., the persons in the videos and audios) was of interest, as this would provide the opportunity to interact with others sharing similar health conditions.

Participants were content with the aesthetics of the website. However, the visual and acoustic quality of the videos and audios could still be improved.

Regarding the functionality of the website, participants mentioned that the website was compatible with various devices such as laptops, desktops, and mobile phones. However, the search engine did not operate correctly, and a few videos were not displayed.

#### Content

Participants agreed that all relevant topics for newly diagnosed persons with MS are presented and that the material was diverse and balanced concerning the selected topics as well as positive and negative experiences.

*“I have an acquaintance who also has MS and I directed him towards this. […] He said he wasn’t sure whether he should take a look because most of the clinics are pro-medication and therefore, they promote it. That wasn’t my impression at all. I told him that and he decided he would contact you and take a look at the website. That was super, really good, I thought. You have a section of people there who have opted against a [drug] therapy.” (person with MS 45)*

Experts also discussed the issue of how to handle obviously false information in the narratives and asked if it might be necessary to add a comment with corrections.

*“If something is misremembered or false information is reproduced, especially on the subject of medication and its active ingredients, I don’t think it would be sufficient clarification to say: “It is possible that some misinterpretations or misunderstandings are contained here that others would regard differently.” […] If it is actually truly wrong, I think it would be better to cut out those parts. […] This could be addressed in the description of the ethical concept of the Website, how care was taken in the videos that if factually misleading information was formulated in the statements, this was taken out. But that otherwise the personal interpretations and evaluations, where there is indeed room for a difference of opinion, were left in.” (expert 2)*

At present, the website only contains experiential information. Some persons with MS asked for links to MS support groups, current research studies and for more detailed information on evidence-based medicine (EbM). Many persons with MS looked forward to the latest patients’ experiences about currently available DMTs or new therapeutic developments (e.g., vaccination against MS, and Covid-19).

For the further development of the website, many people would like to learn more about practical or lifestyle information (e.g., on nutrition and diet, work-life, home design suitable for the disabled) from interviewees. Moreover, patients wished more reports from younger persons. The need to up-date the information on the website was an important point for all participants.

#### Usability

The ease of use is based on attributes such as the architecture of the displayed information, intuitive navigation, reliability and learnability [41]. The architecture of information (see Fig. 1) ensured a convenient operation of the website:

*“I found it easy to navigate, found the layout clear, and I found my way around it with relative ease. You could look for things according to themes or categories and so on. And I found there was clarity here, too. So, really self-evident for someone familiar with searches on the Internet and moving around within it.” (person with MS 12)*

Many found the website easy to navigate and found topics, that they were interested in. However, some also missed certain topics even though they were stored on the website. These illustrate the challenge in designing online information websites, which appeals to all and that the overview of topics and sub-topics could be improved.

The function to adjust the font size points at the developers’ efforts to show awareness for persons with visual impairments. Participants appreciated that the interviews were edited allowing a view at different sub-topics rather than the full interview. They also liked the option to read the transcript in addition to the video or audio.

*“I think that’s a very successful format, because for me the spoken and the visual function conveys a sense of authenticity and makes me feel closer to the person, but with a text in addition for me which I can quickly dip into if I want to work my way through the contents more swiftly.” (person with MS 49)*

Regarding the navigation of the website, users searched for other persons with MS with similar characteristics (e.g., age and sex) and comparable ways of dealing with the disease.

*“Well, I certainly looked for those people of my gender and around my age and who kind of fall into my group […] presuming always that’s where I would find stories similar to my own. Because what is unhelpful for me personally is watching all the catastrophic cases, because I’m not one of them.” (person with MS 12)*

Persons with MS were reassured by the fact that the website was hosted by a credible research institution with a known reputation. Moreover, patients’ experiences were viewed as honest and authentic information.

*“There were very honest reflections, not the producer’s statements, but simply how the individual patients had experienced what was happening with them, why they had stopped taking certain medications and why they had chosen a particular one.” (person with MS 41)*

Persons with MS valued the possibility to access patients’ experiences anonymously, in contrast to other sources of information such as support groups.

#### Satisfaction

#### Gaining emotional support and confidence

Participants liked having access to other people’s experiences online. Patients described that seeing people in similar situations, made the health issues appear more realistic and engaging.

*“I was really surprised for I actually hadn’t expected so many individual videos. […] I think this is something that the disease needs. It is simply the case that there are so many patients with MS where the disease isn’t visible. […] Life can go on quite normally and that is something that a brochure or a basic website cannot get across. Or only in a theoretical way. I thought the lively, intimate form of the videos was great.” (person with MS 48)*

*“What I found really strengthening was how a lot of the people weren’t held back at all, so that you really got the feeling that this could all work out quite differently. Most of them made a really positive impression, the way they spoke, not at all in a pessimistic tone, but rather as though everything will be fine.” (person with MS 41)*

#### Support in decision-making

Persons with MS reported that the website could help newly diagnosed patients to overcome their fears, for example with regard to possible therapies, making them more confident and enabling them to take their time for decision-making.

*“When you are feeling your way as a newcomer, you don’t need to be fearful because there are various possibilities […]. The website leaves everything open, and I really appreciated that, and, most of all, it removes the fear. […] If I’d had a website like this back then, I would definitely have taken a slower approach. […] The diagnosis is something life-changing indeed, but at the end of the day it is not a heart attack. No one is forcing you to act immediately. Your website provides the opportunity to weigh things up, but you don’t have to start straight away, and I believe that’s an important message for those affected.” (person with MS 6)*

Participants claimed that it was helpful to get an overall picture of the different therapeutic options and how other patients decided for or against a particular DMT. Moreover, one patient, for example, described that she was aware of the fact that the effect of a given medication may vary between her and other people on the website.

The interviews showed that the website might be helpful in decision-making for or against DMTs. However, in this respect, the website might appeal more to newly diagnosed MS patients or those who never tried a DMT.

*“For me personally it will most likely be the case that I seldom or never visit the website, because it depends what phase I’m at. I’ve had the diagnosis for over thirty years now.” (person with MS 49)*

The website also opened the eyes to the fact that DMTs may have substantial side effects and that many patients have to switch their DMTs due to these.

*“I found it a bit frightening that lots of patients had those kinds of side effects and that lots had switched from their initial therapy; that many had tried out maybe four or five different therapies. I was taken aback by this content, but it belongs there. It is important that it is told the way it is.” (person with MS 45)*

#### Negative patient experiences perceived as astonishing, and distressing

Some patients stated that they had the impression that the material contains a lot of information about difficulties and constraints. Few missed the ‘joie de vivre’ in the protagonists, especially when they started watching videos on the theme ‘MS diagnosis’. Some people found it difficult to watch and listen to stories that might be overly pessimistic. Participants often rejected experiences that were out forward by individuals whose way of coping with the disease was obviously different from their own.

*“Yes, there is a lot there about the difficulties, if I can put it like that. […] When people recount: “Yes, I had to give up my profession and now I can only work half the time” and that can have a really demotivating effect. […] But I can only say that from my own observational stance. I do think of course there are people who will feel understood by this, who’ll say: “Oh yes, that’s fine to hear there are others having the same difficulties I’m having, and look, that’s how that person dealt with it.” And so, I can’t really judge whether it will be helpful to a lot of people or not. I wouldn’t be enthusiastic about it, but then you can click out of it quickly enough. That’s the nice thing about this format. If you were in a group situation you would have to wait until the person finished talking and listen to everything they had to say.” (person with MS 1)*

Nevertheless, many participants felt that negative experiences should also be part of the website and that the online format allows to leave the potentially discomforting ‘situation’ by simply clicking on the next video or audio. Moreover, no one perceived the videos as too alarming or shocking.

#### Loyalty

Overall, the first impression of the website was positive as it was considered to be reliable, interesting and innovative:

*“It is a very sensible and serious affair. People aren’t depicted in a lurid way as can happen on some television shows.” (person with MS 58)*

*“I was swept along by it, it’s true. […] Yes, it certainly drew me in, and I really did spend hours in front of it.” (person with MS 8)*

Patients wished this website to be accessible more broadly:

*“I would be happy if there was targeted publicity for this in the hospitals and at the neurology practices, with flyers and so on. In my case, diagnosis was not so long ago, and my question then was: What is it like? Where do I find information? I did have a little handbook, which contained quite a lot, but not enough. This homepage provides a chance of covering pretty much every aspect and that is what I needed then.” (person with MS 41)*

## Discussion

### Principal findings

This mixed-methods study aimed to investigate the opinions of persons with MS towards a web-based resource with videos and audios on their experiences, which was developed with systematic methods and active patient involvement.

The evaluation demonstrates that the material on the website covers most of the issues that are important to persons with MS. Besides, the website seems to raise considerable interest as shown by the fact that the mean duration of patients’ sessions visiting the website was 3 hours. Of note, a recent study using a comparable website on colorectal cancer demonstrated that it was only visited for 42 min over a two-week period [18] underlining the huge involvement of the patients in our study.

Most participants considered the PExMS-website easy to navigate, and the information given comprehensible and authentic. As described in a systematic review on risks and benefits of online patients’ narratives, experiential information is a powerful tool to make complex matters more understandable [22, 23]. Besides, the majority judged the website as reliable, since it was developed by a research institution and the material displayed was provided from patients for patients. This has been also shown by other studies evaluating websites with patients’ experiences [16, 42].

There were some discrepancies with regard to the depth of information some persons with MS were looking for. Many participants wished to get “the full picture” (i.e. both the positive and negative experiences) about daily living with MS and the therapeutic options, they were considering, as already described by Synnot, Hill [11]. However, persons with MS often rejected experiences uttered by individuals obviously different from them with regard to age, sex and duration of the disease. Participants often compared their personal situation with that displayed in the online experiences. This finding is in accordance with previous studies dealing with users’ navigation of online patients´ experiences websites [16, 43].

As assessed by the survey, three-quarters perceived the videos and audios as discomforting. However, when asked if the participants considered the website content shocking, they denied it. Some patients explained that they had difficulties to watch and listen to online experiences that were overly pessimistic. One reason for this attitude might be due to the fact that these pessimistic perspectives are in contrast to their own point of view and confront them with a possible future perspective they did not want to consider [12]. On the contrary, persons with MS stated that watching and listening to people in comparable situations had a liberating effect insofar as they felt less abandoned, being part of a community and thus, regained the confidence that their experiences and reactions were normal. However, participants of this study appreciated the option of having access to other patients’ experiences anonymously and without the need for interaction.

Furthermore, persons with MS in our study described variations over time with regard to their behaviour in searching for health information depending on their changing needs, emotional state and growing knowledge about MS. The so-called ‘self-regulation strategies’ affect the extent of how, when and where patients seek health information in response to their prior experience, attitudes, knowledge and their physical or emotional state. In this process of search for information, health literacy is a salient feature for self-regulation [11, 44].

Interviewees uttered that the website might be of help for newly diagnosed patients to overcome their fears, for example with regard to potential therapies, and to get more confident in decision-making. Survey data showed that patients’ confidence to discuss their health condition with others and to communicate with their neurologists at a consultation visit after browsing the website was only moderate. Patients stated to be willing to consult the website if a decision about their health is warranted. Therefore, they perceived the website as helpful when deciding for or against a DMT and felt confident in making an informed choice about a DMT.

More than half of the participants claimed that the website encouraged them to take actions of potential benefit to their health and to play a more active role in their personal healthcare. In line with previous research, patients´ experiences may support other people to recognize decisions which needed careful consideration, to identify and appraise options and to make a choice [17, 45]. Participants studying information on how other patients make decisions result in longer search times for information, thereby increasing their own health literacy [22]. However, using patient experiences may also cause biases in the process of decision-making, since the information format may be randomly generated, unbalanced, taken from a situational context and possibly inaccurate with respect to scientific evidence [22, 23, 46, 47]. Even if experiential information is explicitly presented as an adjunct to EbM information, it may remain unbalanced and might therefore overemphasize potential risks of DMTs as compared to their benefits [22]. On the other side, the transfer of EbM into routine care is often not optimal, as various barriers (i.e., lack of time, available evidence, EbM skills, physician’s attitude, and patient-related factors) that hinder the practice of EbM exist [48, 49]. Moreover, the encouragement of patients to participate actively in the consultation and the decision making is insufficient. For example, according to a survey with *N* = 1500 German patients, only one in four patients experienced shared decision making during primary or specialty care visits within the last 3 years [50]. Thus, the development of patient decision support tools and a continuing education of physicians is needed for a paradigm shift towards a more patient-cantered care [51]. Instead of deriving a clear hierarchy of superiority and inferiority between evidence-based information and experiential information, decision support tools, both factual information and patient experiences should be developed and offered complementary to one another [28].

### Implications for further website development

Although the website was generally viewed positively, several areas of improvement were identified. More specifically, recommendations relating to the web design, appearance and functionality included the improvement of the visual and acoustic quality of the videos and audios, as well as the search engine. The quality of videos and audios is surely depending very much on the circumstances in their production and the quality of the used technical equipment. In the future, these have to be produced more carefully. Moreover, improving or changing the overview of themes and sub-themes may enhance the ease of navigation.

Peer contact is generally appreciated by users of web-based information and interventions [44] and, not surprisingly, some patients asked for an option to interact with the participants whose videos and audios are on the website. The authors of this study, however, deliberately refrained from the implementation of such an interactive tool since it potentially might compromise the persons in the videos and audios.

Recommendations for improvements regarding the content of the website include a detailed check of the accuracy of all medical information provided in the narratives and omission of false factual information in the videos and audios. This does not apply to narratives showing feelings or personal interpretations, since they represent subjective statements in their understanding of the disease and their level of knowledge, which may deviate from scientific information.

Moreover, it was requested to add further experiential information addressing currently available DMTs and new DMTs, as well as information on lifestyle and matters of daily living. As described by Synnot, Hill [11] persons with MS search for practical or lifestyle information to improve their health and for suggestions from other persons with MS on how to manage symptoms. For all participants, the up-to-datedness and the accuracy of the information provided was of critical importance [43], as the internet is considered by most patients as particularly relevant to be kept informed on the latest developments [11, 52], recently and foremost to deal with the Covid-19 crises and the impact of SARS-Cov-2 on persons with MS. Besides, patients were interested to obtain more information on and get access to EbM information. Currently, the website does not provide factual information since the final version of the website will be used in a two-arm pilot randomized controlled trial as a supplement to a website containing EbM information on DMTs [24, 53].

Based on our positive and negative learnings about developing a patient experience website, we’ve created a list of the top 10 key considerations to keep in mind when creating such a website (Table 4).

**Table 4:** Hints for the development of websites with patients’ experiences

Hints for the development of websites with patients’ experiences	
1. Ensure and guarantee neutrality and independence: if possible, avoid an industrial or private company funding. If there is such a funding, guarantee that the sponsor(s) do not have an influence with regard to content. In this case, multi-sponsorship is also recommended. 2. Ensure and guarantee neutrality of the Internet server provider hosting your website: users will expect not to be tracked und connected with third parties. 3. Chose for the right equipment and check your technical devices for the interviews: good video and audio quality are important for the acceptability and a convenient use of the website’s content. Use common formats for interoperability. 4. Try to be exhaustive in your topics and balance the views: ensure that relevant dimensions of your topic are not missing by developing a theoretical sampling strategy in the very beginning. Include the views of patients in the development of topics. Take care that negative and positive experiences on one topic are not drifting in one direction only. 5. Avoid comments or interjections while the interview partner is speaking: interviewer’s expressions like “oh”, “wow” and “m-hm” will be perceived as distracting later in the video or audio. Endure to say nothing in that situation and instead nod silently. 6. Be nice and fair with your interview partners: get their agreement and do not use interview sequences, which are compromising them. 7. Keep authenticity: your interview partners may express views, which are contradicting with your professional knowledge. There is a justifiable reason why and how the persons came to these views and attitudes. These should be represented on the website. Obviously false or misleading information can be cut out in the videos and audios or commented in an amendment. 8. Detailed planning and documentation of decided actions is the key to a successful collaboration: when cooperating with team members on the website, precise agreement, and documentation, e.g., how the videos and audios are to be edited, is particularly important to achieve a certain consistency regarding the design. 9. Develop a reasonable website structure and navigation: make it easy for the users to find quickly the information they find relevant. Implement search and filter functions. 10. Help the users to find further information on evidence-based knowledge and possibilities for help and support: provide information about the disease and the indications or implement relevant links to other related websites, e.g., patient organizations. Think about creating online-forums, chat rooms and/or personal exchanges between users or provide a link to other websites with such possibilities.	

### Limitations

One methodological limitation of the study is that for privacy reasons we did not record log files and whether participants had visited the entire website before completing the survey or participating in the telephone interview or focus groups. The website contains a lot of information, and it would take a significant amount of time (approximately 30 h) to read and to listen to all of it. As a result, participants may have commented only on sections of the website that they had viewed. Moreover, we have to rely on self-reported information, e.g., the mean duration of patients’ sessions visiting the website. Another limitation regarding the interpretation of the usefulness of the website in the process of decision-making for a DMT was the fact that only some patients included in the study were within a decision-making process. Thus, around half of the participants only speculated whether this website might be of help for a decision-making process towards a DMT, since they were not directly affected at the time of the survey. Fourteen of the fifty persons, who had completed the survey, had already taken part in the qualitative interview study in the developmental phase of the website and were thus personally displayed on the website. This fact might have biased the survey results. On the other hand, 13 of 14 patients from the telephone interviews of this evaluation study had not been involved in the developmental phase. Finally, we might have recruited predominantly active information seeking patients which was also reflected in our decisional conflict and empowerment measures. The material might be perceived differently in consecutive patients. Despite these limitations, the study presented here captured a wide range of views from patients and experts. The mixed-methods approach aimed to collect comprehensive responses using different types of data regarding many areas of the PExMS website.

## Conclusion

In summary, the PExMS website was viewed positively, and therefore has the potential to be a useful resource for persons with MS. The findings of this study endorse many concepts of best practice in developing websites with experiential information, such as the importance of good design, visual appeal, and credibility, as well as the presentation of heterogeneous experiences, both negative and positive. However, particular attention must be given to the potential emotional consequences of displaying negative information. Moreover, filter functions for age and sex were important factors of users´ engagement. Patients’ experiences offer both practical and emotional support, but the collection and analysis require intense scrutiny to maintain quality. The results of this study will be used as the basis for subsequent stages of the PExMS project, finally leading to a pilot randomized controlled trial to investigate both usage and the impact of the PExMS-website in the decision-making process for or against a DMT.

## Supplementary Information

**Additional file 1.** Screenshot of the theme ‘DMTs’ on the PExMS-website www.ms-erfahrungen.de. All character names are invented pseudonyms.

**Additional file 2.** Website URL and login details.

**Additional file 3.** Process evaluation questionnaires for patients and experts.

**Additional file 4.** Participants’ quotes on websites’ attributes.

## Data Availability

Qualitative data generated and analysed during the current study are not publicly available due to privacy issues. Quantitative data are available from the corresponding author on reasonable request.

## Abbreviations

CONSORT-EHEALTH: Consolidated Standards of Reporting Trials of Electronic and Mobile HEalth Applications and onLine TeleHealth
DIPEx: Database of Individual Patients’ Experiences
DMT: Disease-modifying therapy
DSES: Decision Self-Efficacy Scale
EbM: Evidence-based medicine
eHIQ: eHealth Impact Questionnaire
MS: Multiple sclerosis
PAM13-D: German Patient Activation Measure 13
PDDS: Patient Determined Disease Steps
PExMS: Patient experiences with multiple sclerosis
PPMS: Primary progressive MS
PrepDM: Preparation for Decision Making scale
RRMS: Relapsing-remitting multiple sclerosis
SPMS: Secondary progressive multiple sclerosis
SURE: Sure of myself; Understand information; Risk-benefit ratio; Encouragement scale
