# Supplementary material for: Development and evaluation of a website with patients experiences of multiple sclerosis: a mixed methods study
Source: BMC Neurol. 2022 Apr 20;22:146. doi: 10.1186/s12883-022-02663-9 (PMC9019288; doi:10.1186/s12883-022-02663-9)
Supplement: Supplementary file 1 — Additional file 1. Screenshot of the theme ‘DMTs’ on the PExMS-website www.ms-erfahrungen.de. All character names are invented pseudonyms. [file 12883_2022_2663_MOESM1_ESM.docx]

#
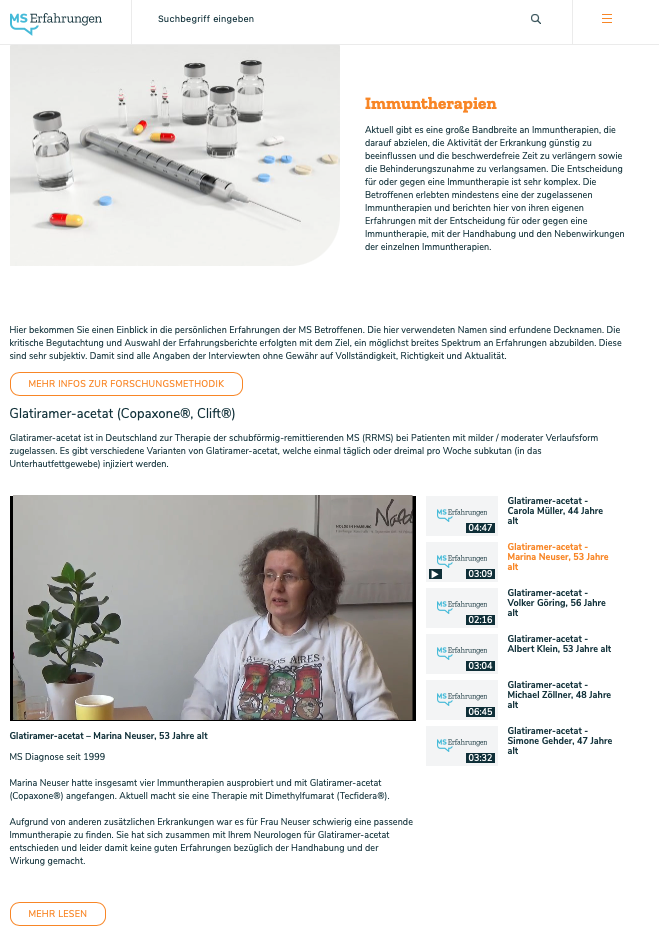


**Translation of the content of the screenshot of the theme**

**‘DMTs’ on the PExMS-website** [**www.ms-erfahrungen.de**](http://www.ms-erfahrungen.de)

**Disease modifying therapies**

Currently, there is a wide range of disease modifying therapies that aim to positively influence the activity of the disease and to prolong the symptom-free period as well as to delay disability progression. The decision for or against a disease modifying therapy is very complex. The people with MS represented here experienced at least one of the approved disease modifying therapies and share their own experiences with the decision for or against a disease modifying therapy, with the handling and the side effects of the individual disease modifying therapies.

Here you will get an insight into the personal experiences of people affected by MS. The names used here are invented acronyms. The critical review and selection of the experiences was done with the aim to represent a broad spectrum of experiences. These are very subjective. Thus, all information provided by the interviewees is without guarantee of completeness, accuracy and timeliness.

More information about the research methods

## Glatiramer-acetat (Copaxone®, Clift®)

Glatiramer acetate is approved in Germany for the treatment of relapsing-remitting MS (RRMS) in patients with a mild/moderate course. There are different versions of glatiramer acetate, which are injected subcutaneously (into the subcutaneous fatty tissue) once a day or three times a week.

[Video]

**Glatiramer acetate - Marina Neuser, 53 years old**

MS diagnosis since 1999

Marina Neuser had tried a total of four immunotherapies and started with glatiramer acetate (Copaxone®). Currently she is on a therapy with dimethyl fumarate (Tecfidera®).

Due to other additional diseases, it was difficult for Ms. Neuser to find a suitable immunotherapy. Together with her neurologist, she decided on glatiramer acetate and unfortunately did not have good experiences with it in terms of handling and effect.

Read more

*On the right: More videos from different interviewees*
