# Supplementary material for: Development and evaluation of a website with patients experiences of multiple sclerosis: a mixed methods study
Source: BMC Neurol. 2022 Apr 20;22:146. doi: 10.1186/s12883-022-02663-9 (PMC9019288; doi:10.1186/s12883-022-02663-9)
Supplement: Supplementary file 2 — Additional file 2. Website URL and login details. [file 12883_2022_2663_MOESM2_ESM.docx]

**Login details to the website:**

[www.ms-erfahrungen.de](http://www.ms-erfahrungen.de)

User name (Nutzername): t.volz

Password (Passwort): MSE-2022!
