# Supplementary material for: Development and evaluation of a website with patients experiences of multiple sclerosis: a mixed methods study
Source: BMC Neurol. 2022 Apr 20;22:146. doi: 10.1186/s12883-022-02663-9 (PMC9019288; doi:10.1186/s12883-022-02663-9)
Supplement: Supplementary file 3 — Additional file 3. Process evaluation questionnaires for patients and experts. [file 12883_2022_2663_MOESM3_ESM.docx]

**Process evaluation questionnaire for patients**

## Which topics (experiences) have you reviewed? (Multiple answers possible)

MS diagnosis

Disease modifying therapies (Therapies)

Lifestyle measures (Therapies)

Rehabilitation, complementary and alternative medicine (Therapies)

Talking about MS (Daily life with MS)

Family and desire to have children (Daily life with MS)

Social life (Daily life with MS)

Employment and education (Daily life with MS)

Holiday planning and travelling (Daily life with MS)

Constrains and adjustments (Daily life with MS)

Dealing with MS

Motivation for participation in the interview

## Were there any topics that you had a broader look at?

No

Yes, MS diagnosis

Yes, disease modifying therapies (Therapies)

Yes, lifestyle measures (Therapies)

Yes, rehabilitation, complementary and alternative medicine (Therapies)

Yes, talking about MS (Daily life with MS)

Yes, family and desire to have children (Daily life with MS)

Yes, social life (Daily life with MS)

Yes, employment and education (Daily life with MS)

Yes, holiday planning and travelling (Daily life with MS)

Yes, constrains and adjustments (Daily life with MS)

Yes, dealing with MS

Yes, motivation for participation in the interview

**Do you consider the website helpful for people with MS in their decision for or against a disease modifying therapy?**

| yes, very | rather helpful | neutral | rather not | not at all |
| --- | --- | --- | --- | --- |
|  |  |  |  |  |

## To what extent did the age of the interviewees influence you in choosing which videos or audios to watch?

| very much |  |  |  |  | not at all |
| --- | --- | --- | --- | --- | --- |
| 1 | 2 | 3 | 4 | 5 | 6 |
|  |  |  |  |  |  |

## To what extent did the gender of the interviewees influence you in choosing which videos or audios to watch?

| very much |  |  |  |  | not at all |
| --- | --- | --- | --- | --- | --- |
| 1 | 2 | 3 | 4 | 5 | 6 |
|  |  |  |  |  |  |

## How often have you logged in to the website within the last four weeks?

More than 1 time a week

Once a week at most

Once a month at most

Never

## How many hours do you have spent approximately on the website in total? _______ hours

## What duration of a video/audio did you find enjoyable to watch/listen to?

a duration of not more than 3 minutes

a duration of 3-6 minutes

a duration of more than 6 minutes

**Were there any technical problems in using www.ms-erfahrungen.de?**

yes

no

If yes, please briefly explain what problems you experienced during the usage:

_______________________________________________________________________

## Do you find that the texts of the website are easy to read due to the customizable font size option?

| yes, very | rather yes | neutral | rather not | not at all |
| --- | --- | --- | --- | --- |
|  |  |  |  |  |

**Which grade would you give the website?**

| excellent | good | satisfactory | adequate | sufficient | insufficient |
| --- | --- | --- | --- | --- | --- |
| 1 | 2 | 3 | 4 | 5 | 6 |
|  |  |  |  |  |  |

## What did you particularly like about www.ms-erfahrungen.de?

____________________________________________________________________________________________________________________________________________________________

## What didn't you particularly like about www.ms-erfahrungen.de?

____________________________________________________________________________________________________________________________________________________________

## What would you like to see in the further development of www.ms-erfahrungen.de?

____________________________________________________________________________________________________________________________________________________________

**Process evaluation questionnaire for experts**

## What topics (experiences) have you reviewed? (Multiple answers possible)

MS diagnosis

Disease modifying therapies (Therapies)

Lifestyle measures (Therapies)

Rehabilitation, complementary and alternative medicine (Therapies)

Talking about MS (Daily life with MS)

Family and desire to have children (Daily life with MS)

Social life (Daily life with MS)

Employment and education (Daily life with MS)

Holiday planning and travelling (Daily life with MS)

Constrains and adjustments (Daily life with MS)

Dealing with MS

Motivation for participation in the interview

## How many hours do you have spent approximately on the website in total? _______ hour(s)

**Did you experience any technical problems while using the website?**

Yes

No

If yes: Please briefly explain what problems you experienced during use:

_______________________________________________________________________

**Did you find it easy to navigate the website?**

| yes, very | rather yes | neutral | rather not | not at all |
| --- | --- | --- | --- | --- |
|  |  |  |  |  |

If rather not/not at all: What can be improved to make it easier to navigate the website?

**Do you think that the texts on the website are easy to read?**

| yes, very | rather yes | neutral | rather not | not at all |
| --- | --- | --- | --- | --- |
|  |  |  |  |  |

If rather not/not at all: What can be improved to make the website texts more easily readable?

**From your point of view, how understandable was the overall content of the website for people with MS?**

| very much understandable |  |  |  |  | not understandable at all |
| --- | --- | --- | --- | --- | --- |
| 1 | 2 | 3 | 4 | 5 | 6 |
|  |  |  |  |  |  |

**How trustworthy did you find the content of the website?**

| very much trustworthy |  |  |  |  | not trustworthy at all |
| --- | --- | --- | --- | --- | --- |
| 1 | 2 | 3 | 4 | 5 | 6 |
|  |  |  |  |  |  |

**Do you consider the website helpful for people with MS in their decision for or against a disease modifying therapy?**

| yes, very | rather helpful | neutral | rather not | not at all |
| --- | --- | --- | --- | --- |
|  |  |  |  |  |

**Does the website give a broad picture of the experience with disease modifying therapies?**

| yes, completely | rather yes | neutral | rather not | not at all |
| --- | --- | --- | --- | --- |
|  |  |  |  |  |

If rather not/not at all: What was missing on the website to more fully represent the experience with disease modifying therapies?

**Which grade would you give the website?**

| excellent | good | satisfactory | adequate | sufficient | insufficient |
| --- | --- | --- | --- | --- | --- |
| 1 | 2 | 3 | 4 | 5 | 6 |
|  |  |  |  |  |  |
