# Supplementary material for: Development and evaluation of a website with patients experiences of multiple sclerosis: a mixed methods study
Source: BMC Neurol. 2022 Apr 20;22:146. doi: 10.1186/s12883-022-02663-9 (PMC9019288; doi:10.1186/s12883-022-02663-9)
Supplement: Supplementary file 4 — Additional file 4. Participants’ quotes on websites’ attributes. [file 12883_2022_2663_MOESM4_ESM.docx]

| **Website attribute** | **Themes** | **Quotes** |
| --- | --- | --- |
| **Web design (use of media, site map, interactivity, search engines), Appearance (layout, font, colors, page length), Functionality (links, speed, compatibility with devices and browsers)** | - Use of different media - Option for forum discussions or to contact the interviewees - Appealing look - Picture and sound quality of the videos/audios could be improved - Compatibility with different devices - Videos from several playlists can be started simultaneously - Search tool does not operate correctly | “Looking through it I found myself wondering at one point, or noting: “Ah, I’d have another question there. I’d like to know more about that.” I’m guessing it’s not possible to contact the people you’ve interviewed? […] What could definitely be of interest for some would be offering a Chat option. I think that perhaps those who were interviewed might also be interested in some exchange or feedback on their statements.” (person with MS 16)  “I perceived the way it was put together very modern, the pictures and the choice of images, all very appropriate. You’ve done really good work there.” (person with MS 41)  “One thing you might consider would be adjusting the volume settings of the various videos. So that they are halfway the same. I’m not sure if different cameras or microphones were in use or perhaps it is the sound produced by the different spaces. Some are relatively soft with something of an echo. Others are relatively clear and direct.” (expert 2)  “I began on the big screen so as to see the films better and so on. It took a little getting used to the scrolling. Later, on my mobile phone the scrolling was very comfortable and deft. That’s when I understood, right, that is the idea behind it.” (person with MS 3)  “There are a couple of little bugs, I think. A technical hitch … the video-link at ‘Alternative methods’, no sign of the video there.” (expert 2)  “The search function, that is totally important. If I have an acute problem, say, and want to find whatever information is available on it, then it would be great if the search function works.” (person with MS 3) |
| **Content (relevancy, completeness, accuracy, timeliness, understandability of the information)** | - Completeness of all relevant topics for newly diagnosed people with MS - Heterogeneity of experiences - Handling false information - Desire for more content and links to evidence-based information (links to self-help organizations, research, EbM information; PEx with vaccination against MS, Covid-19, lifestyle measures, daily life, new DMTs and without DMTs, alternative medicine, rehabilitation and with progressive MS) - Updating of information | “As for the other themes on daily life with the disease, there is a really wide range, I think.” (person with MS 48)  “I also think it’s good that the symptoms and the symptom-therapies are left out. They do make something of an appearance around the edges, in the Living with MS, Lifestyle Factors and so on, but it would be absolutely interminable […] There are an incredible number of symptoms that show themselves in such a variety of ways and intensity and what you can try to get a handle on them. At a certain point, it becomes endless. And then it becomes impossible to find anything.” (person with MS 3)  “I’d say that everything I wanted or needed I found.” (person with MS 38)  “You really can approach the theme of MS in such a variety of ways, and this fact is presented.” (person with MS 6)  “I really liked the mix. Above all how opinion can so strongly differ.” (expert 2)  *“*The person affected said something about Cladribin® that was simply wrong: that it had been available and was then taken off the market because someone died. Perhaps that should be corrected. Otherwise it will stay up there, uncommented upon.” (expert 1)  “I would welcome if the age-range was a little more… I’m relatively young myself, in my early thirties, and there were a lot of people closer to the 50 mark. I would have liked some more younger people because that would hold more interest for me.” (person with MS 45)  “Yes, it is so important that also those persons who are not on an immunotherapy are represented, also that the new drug therapies are presented, too. Though perhaps not immediately after coming on the market. Patients need to gather experience with them first.” (person with MS 3)  “Well, the videos that are there at the moment, maybe every two or three years you’ll have to take a look to see whether they might be changed for others, according to the content. And if things become obsolete, those videos should be replaced, too. I’d welcome that for it would show the people managing the website are staying very up-to-date and that this is more than just a collection point.” (person with MS 12)  “What could also be interesting are current issues. Corona is current and there we have the simple question of whether to be vaccinated or not. That is just one example of current concerns. These interviews are understandably and logically rather general. These current issues would also be really interesting for me. I have read that a vaccination against MS is being worked on at the moment.” (person with MS 45)  “If this is something that can be continued, and if that’s desirable, I think it would be sensible after two or three years once some experience has been garnered [by those with MS], if the new medications could be added in.” (person with MS 3) |
| **Usability (information architecture, intuitiveness, learnability, memorability)** | - Trustworthiness through the research institution as web-developer and the patients as content contributor - Information acquisition as an anonymous - Convenient operation through the information architecture - Better overview of the subtopics necessary - Looking for similarity by age and gender - Barrier-free access | “It is very nice that there is university involvement here.” (person with MS 3)  “I like very much how different individuals speak here in relatively natural surroundings and express their personal take on the themes and how, whether diagnosed some time ago or more recently, one can form a kind of picture of how others are dealing with situations, what their opinions are, without having to partake in the discussion forum of some kind of group-setting.” (person with MS 1)  “I really liked how I could look up clearly defined areas. I didn’t always have to listen to the entire interview and pick out the relevant information, instead it was prepared that way, and I thought that was very good. This way for diagnosis, this way for [drug] therapies, and this way for how I can manage it. [I also found it good] that you’ve written a short summary beneath each video of the person. […] That gives me the chance to say, no, that is not a problem I have, that is not a theme that concerns me anymore. Secondly, I also found it good that thanks to the concise writing I can decide whether to listen to the two and a half minutes or not.” (person with MS 58)  “The area of sport and exercise. I’ve just checked back. And in fact, it is not mentioned and that surprised me somewhat because recently it is featured prominently in other studies and it is often shown how important it is also in MS.” (person with MS 16)  “I have written that I would like it if these points that exist, these sub-sections, if they were immediately visible, and how I really did miss that. I wouldn’t have thought to look under “Everyday Life with MS” for a specific chance to look at, what do I have here for example? There, under “Everyday Life with MS” I find, for example, “Narratives of the Disease”, “A Desire to Have a Child and Family”, “Social Life” and so one. I can click on these points there and find interviews. I’d have found it really helpful if these subsections had been listed additionally at the top, without them being clickable there.” (person with MS 58)  “I naturally looked up the women of my age, that is very interesting and I think that is an automatic reaction. […] I liked that the age was given.“ (person with MS 58)  “I read through the text because I have hearing issues. And the videos were often tricky for me to understand. And then I discovered that they could also be read. That was a good alternative for me.” (person with MS 16)  “I also appreciate the possibility, right at the start, of changing the size of the font, without having to go back to the browser to alter this. That’s really great for it speaks of a high sensitivity also around the theme of ‘accessibility’.” (expert 2) |
| **Satisfaction (usefulness and pleasure)** | - Sense of community - Gaining emotional support and confidence - Support in decision-making - Negative PEx perceived as astonishing, and I.A distressing | “So, I really liked the videos because I’m not alone, that was the feeling I had.” (person with MS 38)  “I discovered all sorts of moments that are very authentic, and something else I liked was how they coincided with my own story. I think that helps in general in the phase when you are out looking, when you are newly diagnosed, to see that you are not alone in the confusion that you encounter in the health system.” (person with MS 49)  “And I definitely took a look at that because I take an immunotherapy, too, and of course I’m always wondering what options I have. […] I appreciated that both sides have a light shone on them […] because different persons with different experiences get a voice. […] I realize, too, that the same medication with the same mechanism of action can have a different effect from person to person. […] But it does no harm to know how other persons are finding it and simply to regard it from a different perspective.” (person with MS 12)  “Of course, there were different opinions, and whether they are mine or not is not the point. There were different age groups, and the younger the patients were, the fewer constraints, or disabilities, they had, of course. So, you couldn’t necessarily identify with this one, or that one, but none of the videos annoyed me or scared me.” (person with MS 41)  “What I generally really liked was having this kind of wide–lens overview. Why people had done something, why they had chosen tablets, why they had decided on a [drug] therapy. Or simply seeing those people, and this was a really positive surprise, who said I have decided against doing anything at all. That is very rare. That was the biggest highlight for me […] seeing how the people are who had opted against doing anything. […] So, seeing these possibilities, realising there are always alternatives … that is so helpful.” (person with MS 50)  “For me personally it will most likely be the case that I seldom or never visit the website, because it depends what phase I’m at. I’ve had the diagnosis for over thirty years now, but I do remember and that’s why I think your project is so fantastic […] that the diagnosis is always a difficult moment. When you think about how everything works it is incredibly difficult to catch hold of a person in the diagnosis phase. To make a long story short: When I received my diagnosis, it was a complex situation. My first step was to go to the Internet and to look for what to do next. I looked for curriculum vitae and wanted to know how those affected dealt with it and what strategies had been developed. […] That very much influenced me in the early years, as to which strategy, which management strategy, and potentially which suggestions, which medical therapies I would try.” (person with MS 49)  “I believe that people who are just starting out are a little overwhelmed by it because it also shows people who have an acute disease course. That is quite uncomfortable for those with a milder course.” (person with MS 50)  “I stumbled a little with it to begin with because I was missing the joie de vivre feel, but that changed completely the deeper in I went. I started with the MS Diagnosis and I wondered why everyone was that way. […] But that wasn’t the case. The people didn’t stay like that. They had different themes, and blossomed, and were really cheery later on.” (person with MS 58)  “There are a couple of people who were not so much my cup of tea because they were very, how should I put it, melancholic. There weren’t many, but there were some. And I didn’t click on them when it came to other subjects because I thought: Oh no, I don’t want to hear the lady on this. […] The people who stood out positively for me were those who were dealing with their illness like I deal with mine: the disease is not my enemy, it’s rather that we live together. It is part of me. I liked watching those people, and didn’t enjoy some of the others. That’s quite normal, every person will find their match on this Website, or positive and helpful suggestions.” (person with MS 48)  “Shocking to me is something that goes beyond what I can imagine, and I didn’t come across that. There were things with a depressive edge […] It could well be that a young person soon after their diagnosis may encounter potentially shocking truths about symptoms.” (person with MS 48) |
| **Loyalty (first impression of the website)** | - Overall positive impression of the website - Desire for greater publicity for this website | “It is a really great project and absolutely pioneering.” (person with MS 1)  “Yes, you can feel how much work has gone into this. I found it exciting to see how open the people were who you were interviewing, I thought that was very interesting.” (expert 4)  “I think it would of course be good for this to be made properly public, so that it reaches people, the people affected, and probably above all those who have been recently affected. I can imagine they would be interested. Thirty years ago, I would definitely have been very interested. […] For example there could be a link to this website on the homepage of the DMSG. That would be no bad thing.” (person with MS 16) |
